# Sleep improves accuracy, but not speed, of generalized motor learning in young and older adults and in individuals with Parkinson’s disease

**DOI:** 10.3389/fnbeh.2024.1466696

**Published:** 2024-09-26

**Authors:** Saar Lanir-Azaria, Rakefet Chishinski, Riva Tauman, Yuval Nir, Nir Giladi

**Affiliations:** ^1^Faculty of Medicine, Tel Aviv University, Tel Aviv, Israel; ^2^The Sieratzki-Sagol Center for Sleep Medicine Research, Neurological Institute, Tel-Aviv Sourasky Medical Center, Tel-Aviv, Israel; ^3^Sagol School of Neuroscience, Tel Aviv University, Tel Aviv, Israel; ^4^Department of Physiology and Pharmacology, Sackler School of Medicine, Tel Aviv University, Tel Aviv, Israel; ^5^Department of Biomedical Engineering, Faculty of Engineering, Tel Aviv University, Tel Aviv, Israel; ^6^Sagol Brain Institute, Tel-Aviv Sourasky Medical Center, Tel-Aviv, Israel

**Keywords:** adaptation, procedural learning, sleep-dependent consolidation, transfer, Parkinson‘s disease

## Abstract

An essential aspect of motor learning is generalizing procedural knowledge to facilitate skill acquisition across diverse conditions. Here, we examined the development of generalized motor learning during initial practice-dependent learning, and how distinct components of learning are consolidated over longer timescales during wakefulness or sleep. In the first experiment, a group of young healthy volunteers engaged in a novel motor sequence task over 36 h in a two-arm experimental design (either morning-evening-morning, or evening-morning-evening) aimed at controlling for circadian confounders. The findings unveiled an immediate, rapid generalization of sequential learning, accompanied by an additional long-timescale performance gain. Sleep modulated accuracy, but not speed, above and beyond equivalent wake intervals. To further elucidate the role of sleep across ages and under neurodegenerative disorders, a second experiment utilized the same task in a group of early-stage, drug-naïve individuals with Parkinson’s disease and in healthy individuals of comparable age. Participants with Parkinson’s disease exhibited comparable performance to their healthy age-matched group with the exception of reduced performance in recalling motor sequences, revealing a disease-related cognitive shortfall. In line with the results found in young subjects, both groups exhibited improved accuracy, but not speed, following a night of sleep. This result emphasizes the role of sleep in skill acquisition and provides a potential framework for deeper investigation of the intricate relationship between sleep, aging, Parkinson’s disease, and motor learning.

## Introduction

Motor learning refers to the ability to acquire new procedural knowledge leading to sustainable improvements in skill performance. Studies of motor learning traditionally rely on paradigms of motor sequence learning, during which a series of simple, stereotyped movements are integrated into a single unitary well-rehearsed sequence (Kami et al., 1995). Following an initial acquisition period, an additional late phase can occur in which the acquired knowledge undergoes further modification during the process of consolidation, resulting in a gain in performance (Censor et al., 2012; Censor, 2013). Importantly, certain aspects of this offline learning are consolidated exclusively during sleep (for review see King et al., 2017). In young adults, task performance is selectively improved across sleeping intervals, while equivalent waking periods confer no such performance benefit (Walker et al., 2002, 2003; Fogel et al., 2014).

A crucial aspect of motor learning is the extent to which learning in a specific setting can be generalized to facilitate the execution of similar skill under novel conditions, without the need to invest time and energy in a new learning process (Censor, 2013). A number of studies have challenged the traditional paradigm and utilized the use of multiple sequences, demonstrating a performance improvement of a novel sequence, associated with a generalization of procedural knowledge formed under the practice of different sequence (Korman et al., 2003; Cohen et al., 2005; Mochizuki-Kawai et al., 2010; Albouy et al., 2013; Song and Cohen, 2014; Friedman and Korman, 2016; Ariani and Diedrichsen, 2019; Lahlou et al., 2022; Johnson et al., 2023). Hence, whereas the use of a single sequence may limit the measured effect to the specific trained movement components comprising a particular sequence, using multiple sequences allows the investigation of intricate procedural learning and its generalization.

An area for further investigation involves the quantification of generalized motor learning (GML) over longer time scales, exploring the role of sleep in the consolidation of generalized knowledge. Though it has been suggested that sleep can help stabilize newly formed memory of GML and facilitate a certain resistance to interference (Korman et al., 2003; Witt et al., 2010; Albouy et al., 2013), the delayed gains in this type of learning were often restricted to sequence-specific elements with different factors postulated to affect the ability to generalize the acquired skill (Korman et al., 2003; Cohen et al., 2005; Witt et al., 2010; Song and Cohen, 2014; Blischke and Malangré, 2017). In particular, the structural and ordinal properties of the trained sequences were shown to engage multiple shifts in the representation of motor experience, mediating different aspects of GML (Korman et al., 2003; Song and Cohen, 2014; Johnson et al., 2023). Likewise, a critical amount of experience with an adequate range of task variations is needed to facilitate the acquisition and transfer of the trained skill beyond sequence-specific elements (Korman et al., 2003; Braun et al., 2009; Yotsumoto et al., 2013). Thus, a thorough examination of GML beyond the boundaries of sequence-specificity requires the utilization of a new practical framework to be implemented in laboratory settings.

Fundamental questions of GML can be further expanded and implemented across ages and under neurodegenerative disorders. Parkinson’s disease (PD) is the most common neurodegenerative movement disorder (De Rijk et al., 1995; Launer et al., 2000; De Lau and Breteler, 2006), involving motor symptoms like rest tremor, muscle rigidity, and bradykinesia, accompanied by non-motor symptoms, including sleep disorders (Jain et al., 2015). Alongside the well-defined clinical symptoms, people with PD often exhibit deficits in motor learning, manifesting as a decrease in the retention of newly learned motor skills, a problem that is present even in the early stages of the disease (Dan et al., 2015). Furthermore, it is of particular interest to investigate sleep-dependent consolidation in patients with PD who exhibit sleep disorders which may further alter consolidation processes.

Previous studies in that field of research have reported inconsistent results (Cristini et al., 2023). Even among studies utilizing homogeneous cohorts of drug-naïve, *de novo* PD patients, it remains unclear whether patients with PD manifest deficits in motor learning and its consolidation (Dan et al., 2015; Marinelli et al., 2017b; Dan et al., 2019; Lahlou et al., 2022). These inconsistencies may be attributed to variations in task properties. Several lines of evidence indicate that while PD patients can acquire procedural knowledge of a single sequence, they are unable to transfer this knowledge into automatic movements (Seidler et al., 2007; Mochizuki-Kawai et al., 2010; Tremblay et al., 2010; Wu et al., 2015; Marinelli et al., 2017a). It has been further postulated that generalized aspects of learning, predominant during the automatization phases, are more susceptible to impairment in PD, as they rely on cortico-striatal motor networks (Doyon et al., 2009; Marinelli et al., 2015; Alm, 2021; Baladron et al., 2023), which are particularly affected by PD, especially during the early stages of the disease (Blesa et al., 2022).

The investigation of GML and its consolidation has not been properly addressed until now in PD. As a first step in our effort to investigate sleep patterns among individuals with PD and their association with motor learning, we broadened the existing paradigms and developed a GML task consisting of multiple sequences. We hypothesized that GML and its consolidation would be impaired in PD patients compared to healthy controls, manifesting as decreased task performance and diminished post-sleep improvement.

To segregate task demands we incorporated a short recall component that followed the standard task blocks. Although memory performance is generally expected to remain intact in the early stages of PD (Evlıce et al., 2021), the introduction of additional cognitive demands can potentially reveal covert cognitive deficits (Yogev et al., 2005; Nieuwhof et al., 2017; Maidan et al., 2019). As has often been the case in similar experimental paradigms, we hypothesize that while young and older healthy adults will maintain intact performance following the additional memory demands, individuals with PD will exhibit a reduction in their performance.

We initially aimed to establish a solid foundation for subsequent comparisons and validate our newly designed task using a gold-standard cohort. To this end, the new tool was employed to examine GML and sleep-dependent consolidation among young, healthy individuals. This was carried out in a two-arms design, aim to control for circadian confounders. Next, the same task was harnessed to explore differences in GML between recently diagnosed, drug-naïve patients with PD and healthy individuals of comparable age.

## Materials and methods

### Participants

Overall, 70 young participants between 20 and 40 years old were recruited for the first experiment of this study. For the second experiment of this study, 30 drugs naïve individuals with a clinical diagnosis of idiopathic PD (H&Y stage 1–2) were recruited from Movement Disorders Clinics of Tel Aviv Medical Centre (TLVMC) and an additional 30 healthy individuals of a similar age were recruited as a control group. Participants had no prior history or presence of neuropsychiatric disorders (with the exception of PD), and did not receive dopamine agonists/ levodopa, antidepressant drugs, antipsychotic drugs, benzodiazepines, or medications that are known to affect wakefulness or sleep. All studies were approved by the TLVMC local human studies committee, and all subjects were informed about the experiment and provided written informed consent prior to any research procedure.

Participants were instructed to refrain from alcohol or caffeine consumption and to avoid taking daytime naps during the 12 h prior to the initiation of the experiment. Screening and initial evaluation comprised cognitive assessment with inclusion criteria including a Montreal Cognitive Assessment (MoCA) score ≥ 23 (Fujiwara et al., 2010), and a Digit Span (DGS) (Jasinski et al., 2011) total test score ≥ 10. The cutoff values were chosen to ensure that cognitive limitations would not hinder participants’ ability to understand and engage in the experiment. In addition to the cut-off on MoCA that has been previously shown to serve as a sensitive measure to detect cognitive impairment (Fujiwara et al., 2010; Freidle et al., 2023), participants with PD also underwent a clinical evaluation by a movement disorders specialist to exclude patients with clinically detectable cognitive decline. Motor evaluation was carried out using the Unified Parkinson’s Disease Rating Scale-Part III (UPDRS-III - motor) (Goetz et al., 2008) with inclusion criteria for healthy individuals of ≤1.

Three subjects were excluded after receiving <23 on the MoCA (0 young, one older control, two PD), and an additional 11 subjects (four young, five older control, two PD), were excluded due to substantial sleep related breathing disorder diagnosed during the experiment. Demographic and clinical details are provided in Table 1.

**Table 1:** Demographics and psychomotor evaluation scores of participants retained for analyses.

	Young control		Older control	PD
	Arm A	Arm B		
Male/female	14/16	12/18	13/24	13/26
Age	31.1 ± 4.6	30.4 ± 3.9	65.0 ± 6.9	64.3 ± 7.1
Digit span total^a^	20.5 ± 3.3	20.2 ± 2.8	16.6 ± 4.0	16.9 ± 4.1
MoCA^b^	28.3 ± 1.5	28.1 ± 1.2	27.8 ± 2.0	27.0 ± 2.0
UPDRS-III^c^	0	0.07 ± 0.2	0.29 ± 0.5^*^	17.6 ± 5.4^*^

Values are presented as mean ± standard deviation. The Wilcoxon rank sum test (Mann–Whitney) was employed for separate comparisons between the two arms of experiment 1 and between the two groups of experiment 2. No significant differences were found in either case, except for a higher UPDRS-III score among the PD group compared to their control group (marked with an asterisk, Z = 6.22, *p* = 0.0000). ^a^Digit Span total = sum of forward and backward span. ^b^MoCA, Montreal cognitive assessment. ^c^UPDRS-III, Unified Parkinson’s Disease rating scale-part III.

### Experimental procedure

#### Experiment 1

**Figure 1:**
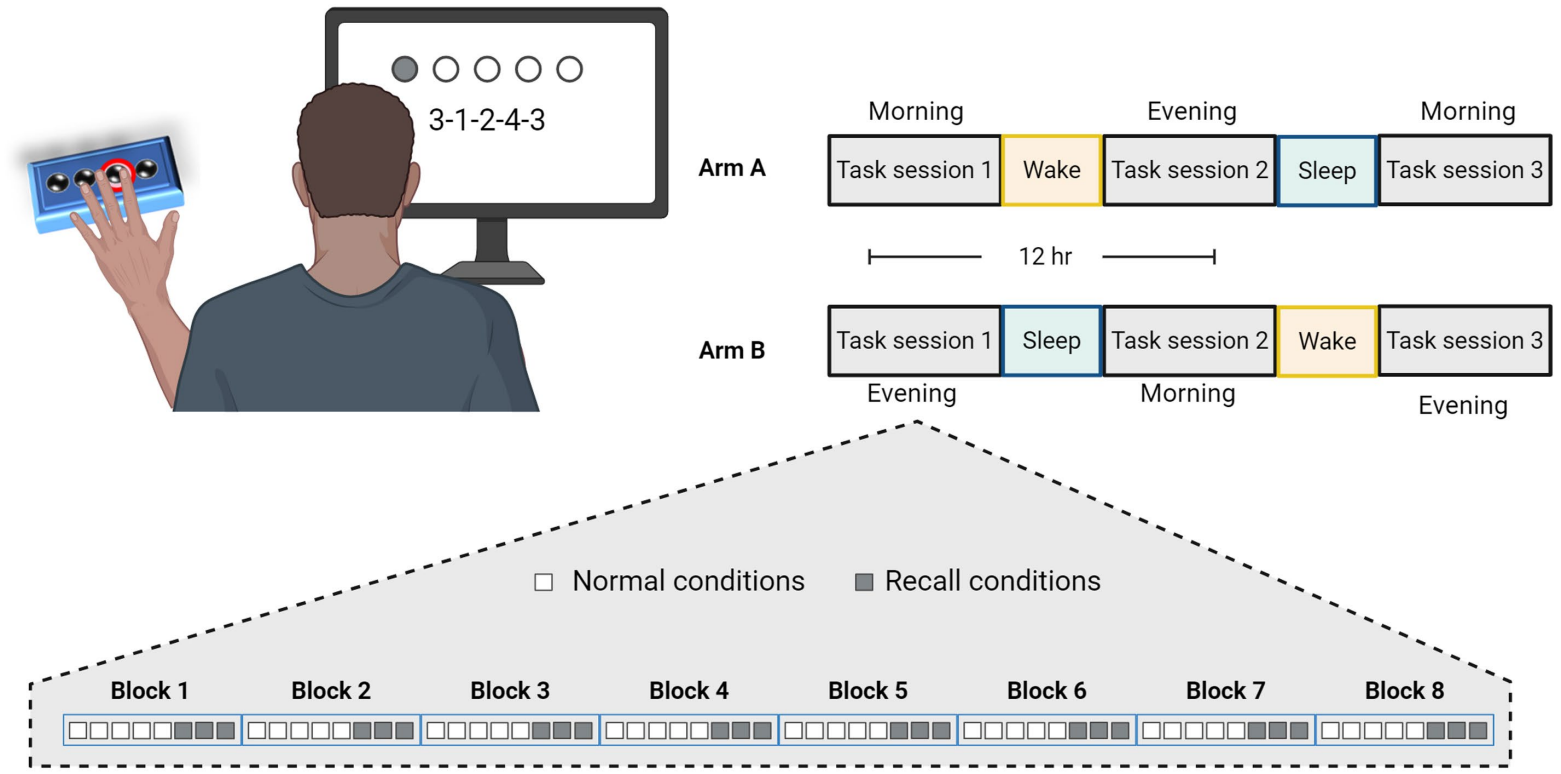
Experimental design and task schedule for experiment 1. Participants were randomly assigned to practice the Generalized Motor Learning (GML) task in three sessions with 12-h intervals, according to two experimental arms, as illustrated in the top right corner. In Arm A, participants performed the first task in the morning, engaged in the second task in the evening after a 12-h wake interval, and performed the third task in the morning following a subsequent 12-h interval that included a night of sleep. Similarly, participants in Arm B performed their first task in the evening, the second task in the morning following a night of sleep, and the third task 12 h later in the evening. Each session consisted of 8 blocks, each comprising a different sequence, with 5 repetitions under normal conditions (i.e., numbers presented on the screen, represented by white squares), followed by an additional 3 repetitions under recall conditions (i.e., repeating the numbers from memory, represented by gray squares).

A total of 60 young subjects were eventually included and were randomly assayed to one of two arms and participated in three GML task sessions performed across 36 h (Figure 1). Arm A comprises a schedule of morning-evening-morning task sessions having participants perform their first task in the morning at a time of their convenience, followed by an evening session 12 h later, and a final session following an additional 12 h that included a night of sleep. Alternatively, Arm B schedule comprises evening-morning-evening task sessions having participants perform their first task in the evening, followed by a morning session 12 h later after a night of sleep, and a final evening session 12 h later. In case sessions schedule was not maintained and the gap between sessions exceeded 13.5 h or fell short of 10.5 h, subject’s data were excluded from the study (six subjects). Overnight sleep time was documented for each subject and averaged across the experimental arms. The average sleep time for participants in Arm A was 6.5 ± 1.4 h, while the average sleep time of participants from Arm B was 6.4 ± 0.8 h, with no significant difference between the two arms (*p* = 0.599).

#### Experiment 2

A total of 26 drug naïve patients with a clinical diagnosis of idiopathic PD and an additional 24 healthy controls of similar age, were eventually included in this part of the study and participated in an overnight experiment. Pre-sleep session was conducted prior to sleep pursued by an additional, post-sleep session in the following morning. Subjects’ sleep was monitored, with older adults spending an average of 7.4 ± 1.9 h in bed, while the PD group spent 7.1 ± 1.4 h in bed. There was no significant difference between the two groups (*p* = 0.336).

The data presented here were collected as part of a broader study and constitute the initial stage in a multi-step research process. Hence, the full experimental procedure involves several modifications compared to the protocol used in experiment 1, including different sleep monitoring approaches and an additional task session performed with participant’s dominant hand.

### Generalized motor learning task

To assess GML beyond the existing confines mentioned above, we developed a self-constructed motor sequential task tailored to measure motor learning while bypassing physical limitations of PD. The task was built as a phyton code generated using PsychoPy 3 (Peirce et al., 2019). It was presented on a standard computer screen with subject’s keypress collected from a response pad with 2–3 millisecond reaction time resolution [Cedrus Corporation (2015), RB-740 Response Pad. San Pedro, California].

The task involves using the non-dominant hand to press four numeric keys and repeating a five-element sequence as quickly and accurately as possible. This requires using a stereotype motor pattern in which each finger corresponds to one number (key) and all fingers aside from the thumb must participate in a synchronized movement.

As illustrated in Figure 1, the task consists of eight blocks each containing a different sequence presented in a random order. Sequences were constructed according to previously used patterns (Kuriyama et al., 2004) and were shown to be equal in difficulty level (Supplementary Figure S1). In order to better adapt the task for people with PD, we incorporated rest periods within each block and reduced the linkage between movement speed and accuracy of performance. Thus, each sequence was presented five times with each repetition interrupted by 2 s rest period. Sequence’s numbers were displayed on the screen (i.e., normal condition) and remained valid with no time limit until five keypresses were obtained. Every keypress produces a white dot on the screen, creating a row from left to right without indicating the specific number typed, to avoid providing precise feedback. Participants were instructed to repeat each sequence as quickly and accurately as possible, and their performance measurements includes separated scores of response time (RT; The time it takes to complete key press, only times of correct responses are included) and the number of correct responses per five-element sequence (CR; the accuracy of performance).

To incorporate an additional cognitive demand and assess the involvement of working memory component, participants were also instructed to retype the same sequence that was just practiced, this time without seeing the numbers on the screen (i.e., recall conditions) for three additional repetitions. Apart from numbers not being presented on the screen, the remaining task properties were left identical under recall conations.

Scores were automatically generated via a self-written code in MATLAB. To avoid trailing error in case of unintended double key press (e.g., instead of 2–4–3-1-2 input = 2–2–4-3-1), the score was blindly corrected to include only the initial error (in the above example input was scored with 4 correct responses out of 5). In any case, such corrections did not exceed 5% of the data.

### Statistical analysis

The calculation of statistical power was conducted as part of a broader multi-step research plan with respect to the overall objectives. Performance scores for the recall condition were calculated separately and independently from the normal performance scores. For each participant, scores were first averaged across individual blocks. Group means were then calculated from individual subjects’ RT and CR scores for each block, as well as the mean session score averaged across all eight session blocks.

To investigate within-session improvement and distinguish it from longer-timescale processes, we focused on the first session. Specifically, we analyzed the difference between two edge points: the first two blocks and the last two blocks of that session. To encompass overall learning, we calculated the difference in performance between a third time point, comprising the last two blocks of the final session, contrasted against the first two blocks of the first session. Finally, between-session improvement was quantified as the difference between two consecutive sessions, with each session’s score averaged across all eight blocks. The effect of increased memory load under sequence recall conditions was assessed by comparing the mean session scores between normal and recall conditions.

Data processing and analysis were carried out using MATLAB (The MathWorks Inc., Natick, MA, United States) and figures were further edited with BioRender.com. Non-parametric tests were chosen following data distribution testing with a one-sample Kolmogorov–Smirnov test, which indicated that the data were not normally distributed. Within arm/group differences were evaluated using Wilcoxon signed-ranks test, whereas between arms/groups differences were evaluated using Wilcoxon rank sum test (Mann–Whitney). We employed two-tailed tests with statistical significance set at an alpha level of 0.05. To assess the potential effect of age and group on post-sleep improvement, we divided participants in both the PD and control groups into two age sub-groups (above and below 65 years), and applied a bootstrap-based, non-parametric two-way ANOVA test with 10,000 iterations. Correlations between motor and cognitive assessment scores and task performance were tested using Spearman’s rank correlation test. We corrected for multiple comparisons using the Bonferroni method. In either case, a raw *p*-value was reported but it was stated as significant only if it survived the correction. Data are presented in the text as mean values ± standard deviation (SD). Data are presented in the text as mean values ± standard deviation (SD).

## Results

To characterize GML and assess the contribution of sleep to the consolidation of the newly obtained procedural knowledge, healthy young volunteers participated in experiment 1, as described in detail in Figure 1. Subjects in arm A followed a morning-evening-morning schedule and performed their first task at the morning at a time of their convenience (*n* = 30, mean hour 9:01 AM ±89 min), whereas subjects in arm B followed an evening-morning-evening schedule and performed their first task at the evening (*n* = 30, mean hour 8:46 PM ± 110 min).

**Figure 2:**
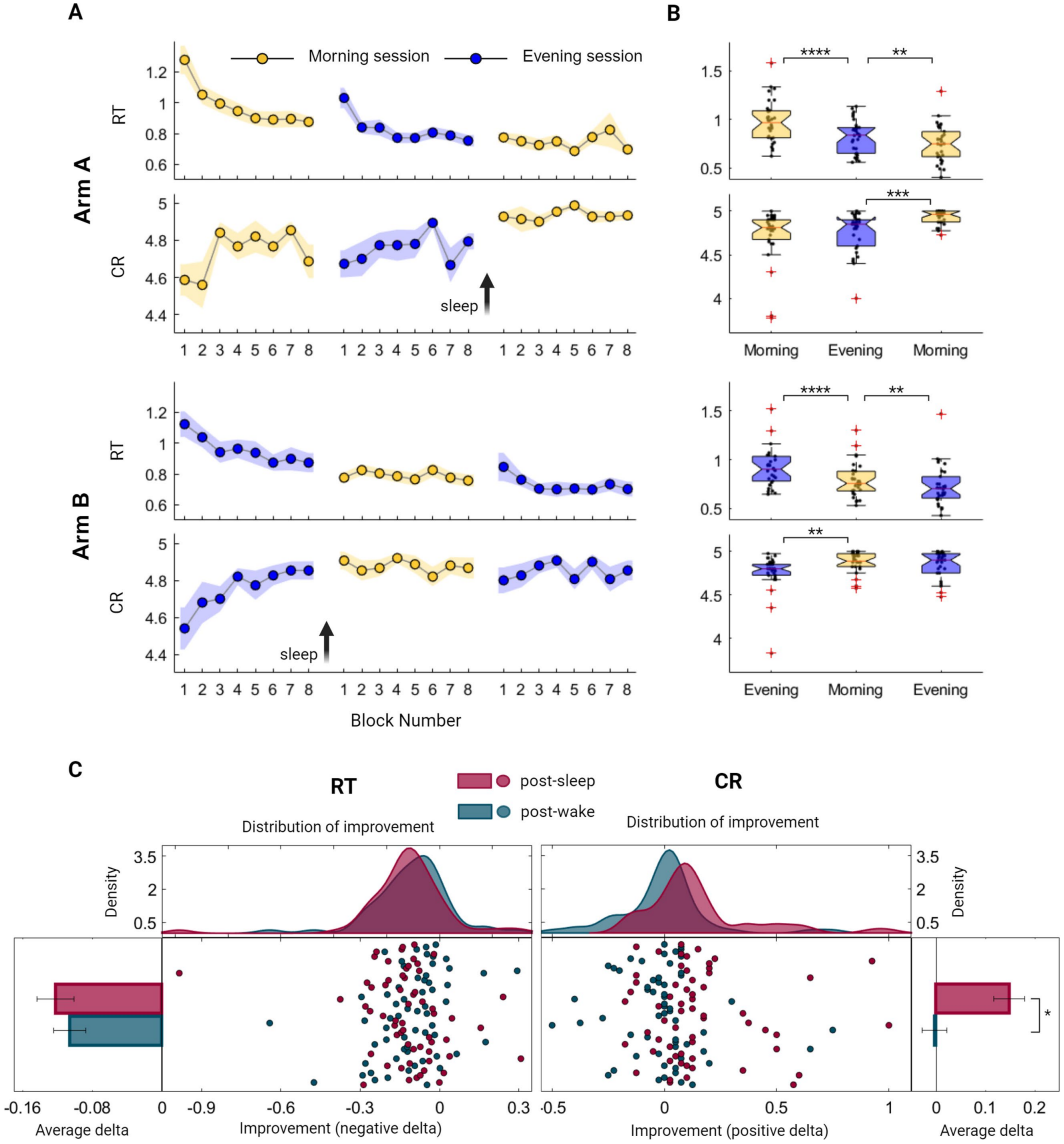
Evaluation of Generalized Motor Learning (GML) among healthy young individuals throughout the sleep–wake cycle. **(A)** Group averages of Reaction Time (RT; the time in seconds it takes to complete key press) and Correct Responses (CR; the number of correct responses per five-element sequence) across morning blocks (yellow) and evening blocks (blue) of participants from arm A (upper panel) and participants from arm B (lower panel). The standard error of the mean (SEM) is represented by a shaded area, while arrows are used to indicate the time of the sleep interval. **(B)** Boxplots portraying the median, the 25th and 75th quartile values of RT and CR averaged across task blocks during the three task sessions of participants from arm A (upper panel) and arm B (lower panel). Individual performance measurements are indicated as black circles, while red crosses denote outliers, and whiskers extend to non-outlier extreme values. **(C)** Individual improvement indexes were quantified as the difference between the mean scores of two consecutive sessions, with either sleep or wake intervals separating them [i.e., post-sleep (maroon) or post-wake (turquoise) respectively]. These indexes merged across participants of both arms, are presented as mean ± SEM of RT (left panel) and CR (right panel). Improvement in speed encompasses negative values, further represented as individual indexes with fitted kernel distributions plotted above (left-middle panel), capturing similar curves of the post-wake and post-sleep improved RT. Analogously, accuracy improvement consists of CR positive values, represented as individual indexes with fitted kernel distributions (right-middle panel), indicating certain incongruity between the two curves. The performance scores presented in this figure include those obtained under normal condition (i.e., without additional cognitive demand) and do not include scores from the recall condition. Significant differences are marked, *p < 0.05, **p < 0.005, ***p < 0.0005, ****p < 0.00005.

We initially explored whether an immediate, rapid generalization of sequential learning and a transfer of procedural knowledge from one sequence to another could be observed in our data. To quantify this short-timescale GML, we focused on within-session improvement under normal task condition (i.e., without additional cognitive demand) examining initial session blocks where longer time-scale consolidation processes are yet to exist. Indeed, a marked stepwise improvement is clearly evident during the first session in both arms (Figure 2A). This practice-mediated GML can be deduced by measuring the difference in performance between the first two blocks and the last two blocks of the first session. Participants in arm A demonstrated an enhancement in performance, with an average speed improvement of 19.5 ± 22.6% (Z = 3.84, *p* = 0.000) and accuracy improvement of 5.0 ± 9.9% (CR; Z = 2.64, *p* = 0.008). Similarly, participants in arm B exhibited an average speed improvement of 15.7 ± 27.9% (Z = 3.73, *p* = 0.000) and an accuracy improvement of 8.7 ± 29.9% (Z = 2.5, *p* = 0.011). There were no significant differences between the two arms when comparing first-session performances (Z^RT^ = 1.06, P^RT^ = 0.290; Z^CR^ = 0.61, P^CR^ = 0.538), or the extent of first-session improvement (Z^RT^ = 0.18, P^RT^ = 0.853; Z^CR^ = 0.44, P^CR^ = 0.662), indicating that the time of the day had no significant effect on performance.

Next, a global overall performance gain was calculated to assess the maximal learning capacity across the experiment. Participants in both arms exhibited similar learning patterns, with no significant differences observed between the groups (Z^RT^ = 0.64, P^RT^ = 0.520; Z^CR^ = 1.46, P^CR^ = 0.072). This evaluation demonstrated an enhancement in performance, with an average reduction in RT of 0.4 ± 0.2 s, corresponding to a speed improvement of 33.0 ± 15.3% (Z = 6.73, *p* = 0.000). Similarly, the average CR increased from 4.6 to 4.9, which represents an accuracy improvement of 8.6 ± 22.5% (Z = 4.16, *p* = 0.000).

Although both speed and accuracy measurements demonstrated the existence of long-timescale performance gains, summarizing group scores across the sleep–wake cycle reveals distinct modulation processes. While speed consistently improved when comparing each session to the preceding one, this enhancement was notable across all task sessions, regardless of the sleep–wake interval (Figure 2B; *p* < 0.005 across all intervals). In contrast, accuracy was exclusively modulated in a sleep-dependent manner, presenting a significant improvement following a sleep interval but not after a wake interval (Figure 2B). Individual variations in the extent of improvement were not explained by cognitive assessment scores (Spearman’s rank correlation, *p* > 0.05; see also Supplementary Figure S3).We further explored trends in performance gains by computing individual delta values following wake and sleep intervals in both arms. As illustrated in Figure 2C, there was no significant difference in the extent of speed gain following wake and sleep intervals (Z^RT^ = 0.87, P^RT^ = 0.385), with individual improved delta values exhibiting similar distribution curves under these two conditions. In contrast, accuracy gain is significantly larger following sleep interval (Z^CR^ = 4.32, P^CR^ = 0.000), reflected as a deviation of the distribution curve toward positive values. Importantly, increasing memory load under sequence recall conditions resulted in improved speed (mean RT decrease across all sessions 32.4 ± 11.5%, Z_arm A_ = 4.782, P_arm A_ = 0.000; Z_arm B_ = 4.782, P_arm B_ = 0.000) without a decrease in accuracy levels (mean CR decrease across all sessions 1.3 ± 5.2%, Z_arm A_ = 0.05, P_arm A_ = 0.959; Z_arm B_ = 1.30, P_arm B_ = 0.190, see also Supplementary Figure S2 and Table S1), indicating that accuracy measurement represents a relatively pure procedural trait.

**Figure 3:**
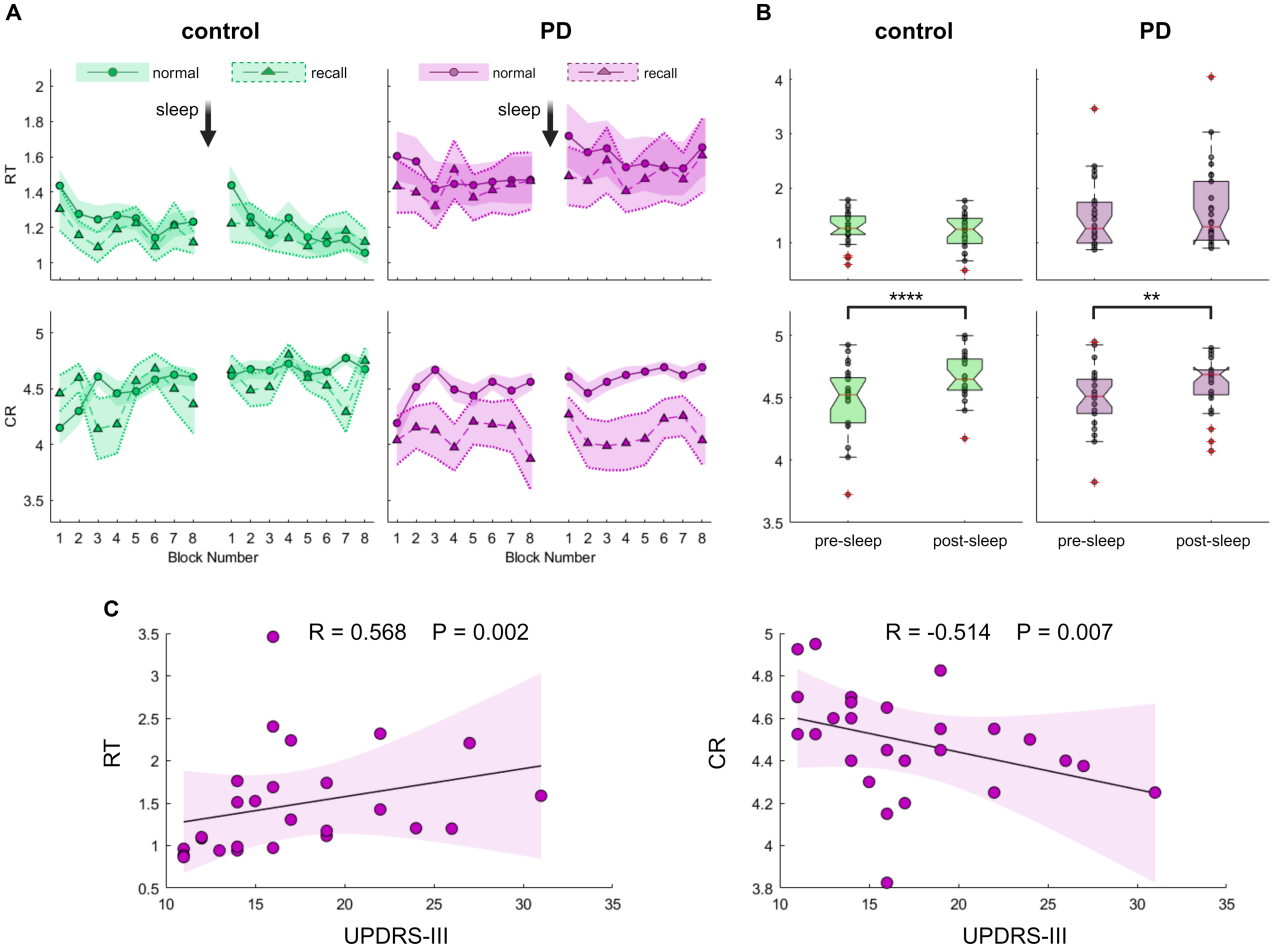
Evaluation of Generalized Motor Learning (GML) among patients with Parkinson’s disease (PD) and healthy individuals of comparable age. **(A)** Blocks averages of Reaction Time (RT; the time in seconds it takes to complete key press, upper panel) and Correct Responses (CR; the number of correct responses per five-element sequence, lower panel) under recall (triangles) and normal (circles) task conditions for the PD group (purple, right panel) and the control group (green, left panel). The Standard Error of the Mean (SEM) is displayed as a shaded area with a dashed line on its border across recall blocks for better visibility. **(B)** Boxplots depicting the median, the 25th, and 75th quartile values of RT (upper panel) and CR (lower panel) averaged across normal condition task blocks during pre-sleep and post-sleep task sessions for the control group (left panel, green) and the PD group (right panel, purple). Individual performance measurements are indicated as circles, while red crosses denote outliers, and whiskers extend to non-outlier extreme values. Significant differences are marked, *p < 0.05, **p < 0.005, ***p < 0.0005, ****p < 0.00005. **(C)** Correlation of PD group task scores obtained during the pre-sleep session, under normal task conditions plotted against UPDRS-III (Unified Parkinson’s Disease Rating Scale-Part III). Individual performance measurements of RT (left panel) and CR (right panel) are shown as circles, with the solid black line representing the linear regression, and the shaded area indicating the 99% confidence interval. Spearman’s rank correlation test shows a significant correlation, emphasizing the association between poorer task performance and more severe motor symptoms. The *p*-value cutoff, following adjustment for multiple comparisons, is 0.0125.

Having shown that the new task is a validated tool to assess GML among young individuals, we were now able to further explore this process in the occurrence of PD, focusing on overnight performance gain. For this aim, 26 drug-naïve individuals with recently diagnosed PD and 24 healthy controls of similar age, participated in experiment 2 comprised task performance prior to sleep and immediately following awakening. Summarized group performances are illustrated in Figures 3A,B. Although the average RT of the PD group was higher compared to the control group (RT_PD_ = 1.5 ± 0.7 s, RT_control_ = 1.2 ± 0.3 s), this difference was not statistically significant (Z = 0.98, *p* = 0.330). Likewise, there were no significant group differences in mean accuracy level (CR_control_ = 4.58 ± 0.24, CR_PD_ = 4.55 ± 0.23, Z = 0.31, *p* = 0.754). Nevertheless, lower performance among individuals with PD appears to be associated with the severity of motor symptoms. As shown in Figure 3B, both speed and accuracy were significantly correlated with UPDRS-III scores (R^RT^ = 0.568, P^RT^ = 0.002; R^CR^ = −0.514, P^CR^ = 0.007).

To assess performance gain following a night of sleep, we quantified the difference in task performance between the two sessions. In a continuous line with the results described above in young subjects that differentiate the modulation processes of the two task components, both control and PD groups exhibited no significant post-sleep improvement in speed (Figure 3B, Z_control_ = 1. 46, P_control_ = 0.152; Z_PD_ = 1.05, P_PD_ = 0.303) but displayed a significant post-sleep improvement in accuracy (Z_control_ = 4.06, P_control_ = 0.000; Z_PD_ = 2.86, P_PD_ = 0.003). Healthy control subjects exhibited an average post-sleep accuracy improvement of 4.7 ± 4.0% whereas participants from the PD group exhibited an average increase of 2.9 ± 4.5%. Although the results suggest a trend toward diminished post-sleep improvement in the PD group, this trend did not reach statistical significance (Z = 1.89, *p* = 0.058). We further tested for a possible effect of age and group using a bootstrap-based, non-parametric two-way ANOVA test and found no significant effects of age on speed improvement (*p* = 0.382) or accuracy improvement (*p* = 0.731). We also excluded potential interactions between cognitive assessment scores and post-sleep improvement that did not reach a significant level after correcting for multiple comparisons (Supplementary Figure S4). Although there was a significant correlation between the severity of motor symptoms in PD patients and initial task performance, this relationship did not retain its significance when tested against post-sleep improvement (Spearman’s rank correlation test between UPDRS-III and post-sleep improvement: R^RT^ = -0.083 P^RT^ = 0.686; R^CR^ = -0.108 P^CR^ = 0.599).

**Figure 4:**
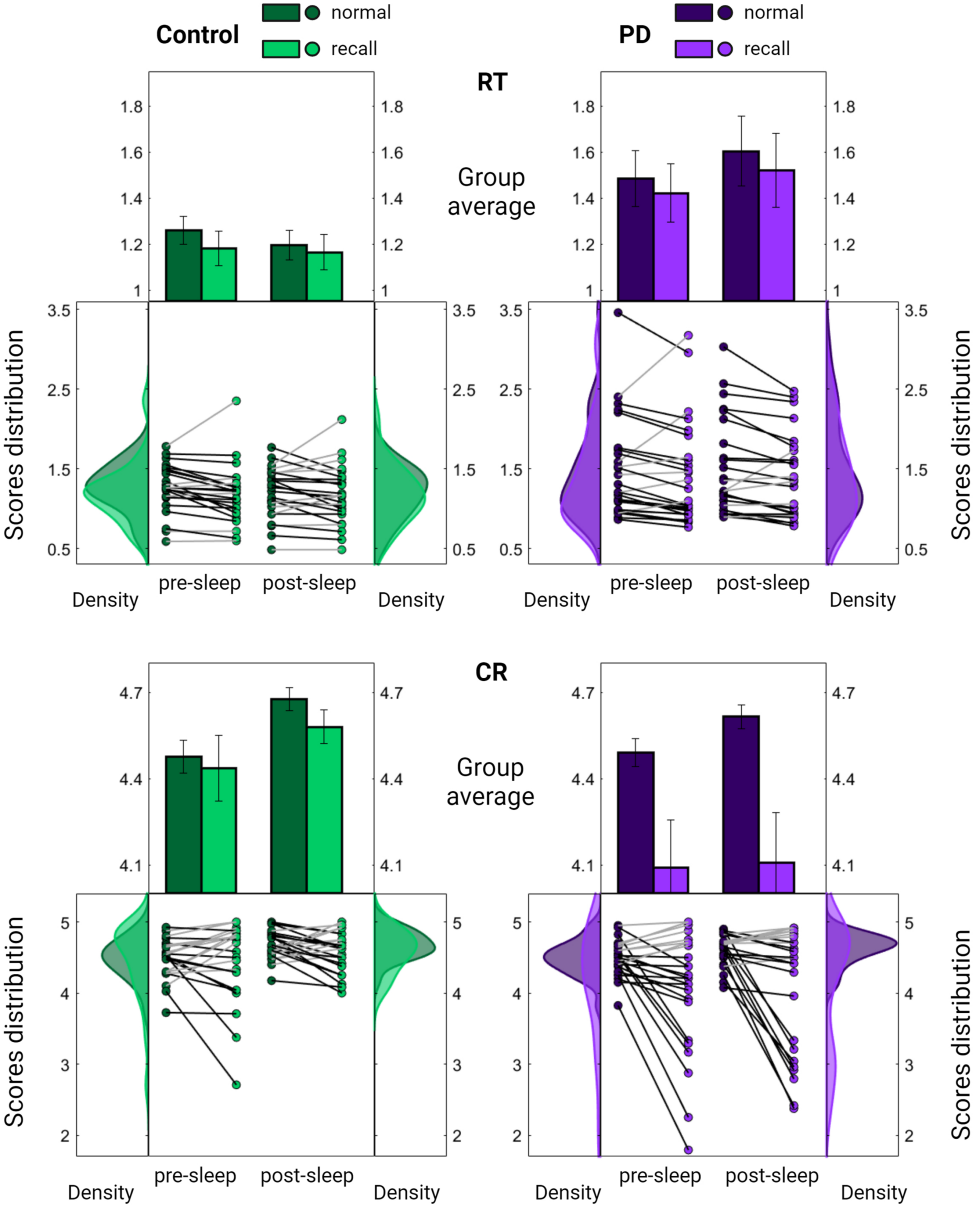
Comparison of task performance under normal and recall conditions. Summarized evaluation of Reaction Time (RT; the time in seconds it takes to complete key press, upper panel) and Correct Responses (CR; the number of correct responses per five-element sequence, lower panel) among the PD group (purple, right panel) and the control group (green, left panel) across pre-sleep and post-sleep sessions. In each subplot, the middle section displays individual mean scores under normal (darker color) and recall (lighter color) conditions averaged across task blocks. Lines connect each subject’s scores under the two conditions, with black lines indicating a decline in performance. Results are further summarized as mean ± SEM in the upper section with distribution curves presented in the right and left sections. Significant differences are marked, *p < 0.05, **p < 0.005.

Nonetheless, upon examining group performances under sequence recall conditions, a notable distinction emerged. While individuals in the control group displayed a pattern akin to that observed in young participants, maintaining accuracy level under sequence recall condition (Figures 3A, 4; mean decrease across all sessions 1.5 ± 7.5%, Z = 0.84, *p* = 0.399), individuals with PD exhibited a significant and consistent decline of 10% in the number of correct responses across recall blocks (mean decrease across all session 10.3 ± 16.2%, Z = 2.37, *p* = 0.018). The reduction in accuracy was accompanied by certain improvement in speed (mean increase across all sessions 4.8–5.3 ± 11.6–11.8%, Z = 2.34–2.43, *p* = 0.019–0.015 for control and PD groups respectively), perhaps indicating an accuracy-speed trade-off. Although this possibility cannot be ruled out, there was no significant difference in speed improvement between the groups (comparing the overall delta between normal and recall RTs; Z = 1.21, *p* = 0.226), with both groups showing highly overlapping RT distribution curves under normal and recall conditions (Figure 4, upper panel).

Conversely, there was a significant group difference in the decrease of accuracy under recall conditions (Z = 2.00, *p* = 0.045), with the PD group showing a deviation from the distribution obtained under normal conditions (Figure 4, lower panel).

Importantly, no significant differences were observed between the groups in cognitive assessment scores (Table 1; P^MoCA^ = 0.708 P^DGS^ = 0.988). Furthermore, the decrease in accuracy under recall conditions was correlated with the severity of motor symptoms (R^CR^ = 0.440, P^CR^ = 0.024) whereas no such correlation was found with the improvement in speed (R^RT^ = 0.178, P^RT^ = 0.385). Taken together, these findings suggest that the altered accuracy pattern may reflect a disease-related abnormality.

## Discussion

This study was conducted as part of a multi-stage research plan aimed at elucidating the contribution of sleep to motor performance in individuals with PD and healthy individuals across life stages. An essential contribution of this work is the development of a newly designed motor task, which has been shown to provide a valid evaluation of GML. Furthermore, the results presented herein demonstrate generalization of sequential learning through multiple learning-related phases. An immediate, rapid generalization of sequential learning and a transfer of procedural knowledge from one sequence to another was reported in young healthy subjects. A secondary longer timescale performance gain was also observed, wherein sleep facilitated improvement in accuracy, but not in speed, across all groups. Subjects in the PD group demonstrated comparable performance abilities to their equivalent age group in most measurements. However, distinctively, they were unable to sustain their accuracy level when confronted with additional memory demands during sequence recall conditions.

In conjunction with similar reports, our observations reveal that different components of generalization develop over a diverse range of timescales, from seconds to hours and days (Bönstrup et al., 2019, 2020; Gupta and Rickard, 2024). The initial learning phase reported here among young healthy volunteers encompasses a swift enhancement in both speed and accuracy detected within a single session, aligning with the model of rapid learning processes that contribute to early skill acquisition over a short timescale (Bönstrup et al., 2019, 2020; Johnson et al., 2023; Gupta and Rickard, 2024). Our observation is consistent with previous research demonstrating a rapid gain in performance resulting from the transfer of knowledge acquired during the practice of one sequence, applied to the execution of a novel one (Korman et al., 2003; Ariani and Diedrichsen, 2019; Johnson et al., 2023). This early learning process constitutes a rapid form of consolidation (Bönstrup et al., 2019, 2020), related to changes in memory circuits (Chen et al., 2015), underpinned by rapid wakeful neural replay (Jacobacci et al., 2020; Buch et al., 2021).

Although the primary representation of generalized task knowledge provides a possible substrate for GML, additional time can further stabilize the newly formed memory (Robertson, 2018).This is also supported by the delayed gain in performance reported here across ages and among both healthy individuals and patients with PD. The two-arm design implemented among young adults revealed two distinct consolidation processes, each contributing to different aspects of GML. Specifically, speed of movement exhibited a delayed improvement extending over a longer time scale but appeared to be modulated by both practice and time, whereas accuracy showed a strong reliance on sleep, conforming to the classical model of sleep-dependent consolidation. Despite previous indications for the occurrence of sleep-dependent consolidation of GML (Korman et al., 2003; Witt et al., 2010; Meier and Cock, 2014; Song and Cohen, 2014), accuracy has not been previously considered as the prime quantitative marker to facilitate this knowledge shift. This is similar to reports from non-generalized motor learning experiments, where the lack of accuracy improvement was explained by the fact that participants often demonstrated a ceiling effect, having no room for practice-induced improvement. Whereas most of these studies rely on a single sequence and thus limit the measured effect, our approach incorporated multiple sequences beyond what was previously tested, expanding the range of potential outcomes. Noteworthy, the presence of a slow, sleep-dependent accuracy enhancement is reported here across young adults, older adults, and individuals with PD, emphasizing its universal nature.

The absence of a significant difference between the PD and control groups is particularly surprising and contradicts our initial hypothesis. Given the involvement of striatal motor networks in generalized aspects of learning (Doyon et al., 2009; Marinelli et al., 2015; Alm, 2021; Baladron et al., 2023), we anticipated lower performance among individuals with PD compared to older controls.

Conversely, our results demonstrating overnight stabilization of generalized motor memory in both groups suggest that early-stage naive PD patients, at least to some extent, retain the ability to learn and consolidate procedural knowledge. However, the observed correlation between motor-symptoms severity and poor task performance indicates that disease-related mechanisms are indeed at play. Furthermore, although the group differences did not reach statistical significance, our data showed a clear trend of reduced performance in the PD group. It is plausible that a larger sample size and the inclusion of more severely affected PD patients would reveal significant differences.

Notably, our cohort consisted of drug-naïve, early-stage PD patients, raising the question of whether the absence of differences in GML would persist in later stages of the disease or would be modulated under pharmacologic dopamine replacement. Furthermore, due to variations in experimental design, we did not compare the performance scores of young healthy individuals with those of PD patients and healthy older adults. Thus, we cannot exclude the possibility of an age-related GML deficit common to both older individuals with and without PD.

Nevertheless, despite the comparable accuracy level of control and PD groups across task sessions, a substantial difference was observed under sequence recall conditions, where an additional memory demand was incorporated. Given that the recall condition occurred immediately after five repetitions of visually guided normal trials, it likely reflects short-term memory retention of the sequence. However, cognitive assessments revealed no significant differences between the groups in this or other cognitive domains. The significant decline in accuracy among individuals with PD might relate to the fact that they are more susceptible to interference in performance of motor tasks when paired with simultaneous cognitive demand (dual tasking) (Yogev et al., 2005; Nieuwhof et al., 2017; Maidan et al., 2019). Having less available cerebral resources to rely upon, PD patients often experience a cognitive overload when facing a dual-task paradigm (Yogev et al., 2005; Nieuwhof et al., 2017; Maidan et al., 2019). An alternative explanation may lie in the fact that the normal task conditions provide participants with exterior cues that facilitate externally triggered movements, prominently activating the cortico-cerebellar network (Gowen and Miall, 2007; Purzner et al., 2007; Taniwaki et al., 2013; Huang et al., 2019). In contrast, recall conditions require the utilization of internally guided movements, which preferentially involve the cortico-basal ganglia network (Purzner et al., 2007; Vaillancourt et al., 2007; Filyushkina et al., 2019; Huang et al., 2019). It has been proposed that individuals with PD are prone to motor deficits when relying on internal cues due to their dysfunctional cortico-basal ganglia network and that motor performance can be overcome by external cues since the cortico-cerebellar network remains intact (Jahanshahi et al., 1995; Lewis et al., 2007; Petrucci et al., 2022). The GML task’s competence to detect this behavioral relapse signifies its potential for investigating mechanisms of motor control and their involvement in PD.

In conclusion, this study has demonstrated the acquisition and consolidation of generalized motor knowledge across multiple learning phases. The research primarily focuses on general skill learning and did not specifically quantify sequence-specific learning. While sequence-specific learning may play a role in motor performance improvements, the experimental design and analysis were not tailored to directly measure these effects. Further investigation is needed to isolate and examine the distinct contributions of sequence-specific learning within this framework. General learning patterns were consistently observed across different ages, health and diseases, and encompass gains in performance facilitated by sleep, emphasizing the universal nature of sleep as a beneficial factor that stabilizes and sustains newly acquired skills. Further research is needed to investigate the contribution of specific sleep components to the consolidation of GML. Given the observed decline in the performance of individuals with PD under sequence recall conditions, a promising avenue for future research involves expanding this investigation and delving into the underlying mechanisms that contribute to this phenomenon.

## Data Availability

The raw data supporting the conclusions of this article will be made available by the authors, without undue reservation.
