# Supplementary material for: Sleep improves accuracy, but not speed, of generalized motor learning in young and older adults and in individuals with Parkinson’s disease
Source: Front Behav Neurosci. 2024 Sep 26;18:1466696. doi: 10.3389/fnbeh.2024.1466696 (PMC11464313; doi:10.3389/fnbeh.2024.1466696)
Supplement: Supplementary file 2 [file Data_Sheet_2.pdf]

Supplementary Material

1 Supplementary Figures and Tables

1.1 Supplementary Figures

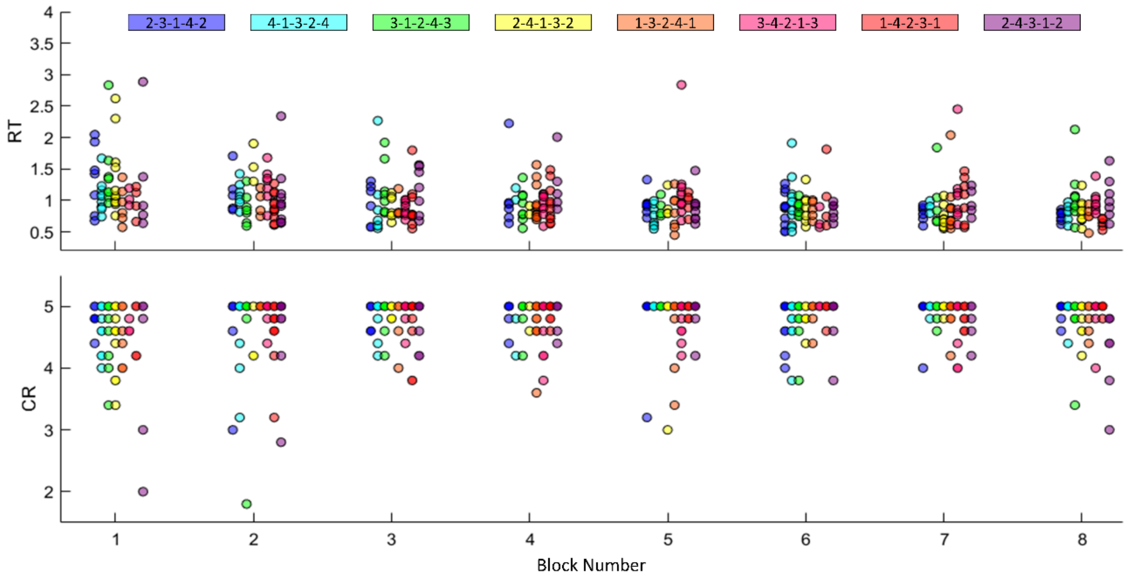

Figure S1. Comparison of task performance under different sequences. Each task block comprises a single sequence randomly assigned to that block. Participants were assigned the same sequences but in a different order. Individual scores of Reaction Time (RT; the time in seconds it takes to complete key press, upper panel) and Correct Response (CR; the number of correct responses per five-element sequence, lower panel) of young participants from the two experimental arms are presented as colored circles across blocks obtained in the first session. Block scores are subdivided into different sequences and color-coded accordingly, with the legend indicating the exact sequences. To address visibility concerns arising from overlap due to identical scores, particularly frequent in the case of CR, individual score circles are semi-transparent, facilitating color accumulation to indicate such overlap. All sequences consist of a constant number at the beginning and end, with different numbers in between. No performance benefits or downfalls were found for either sequence ($P > 0.05$).

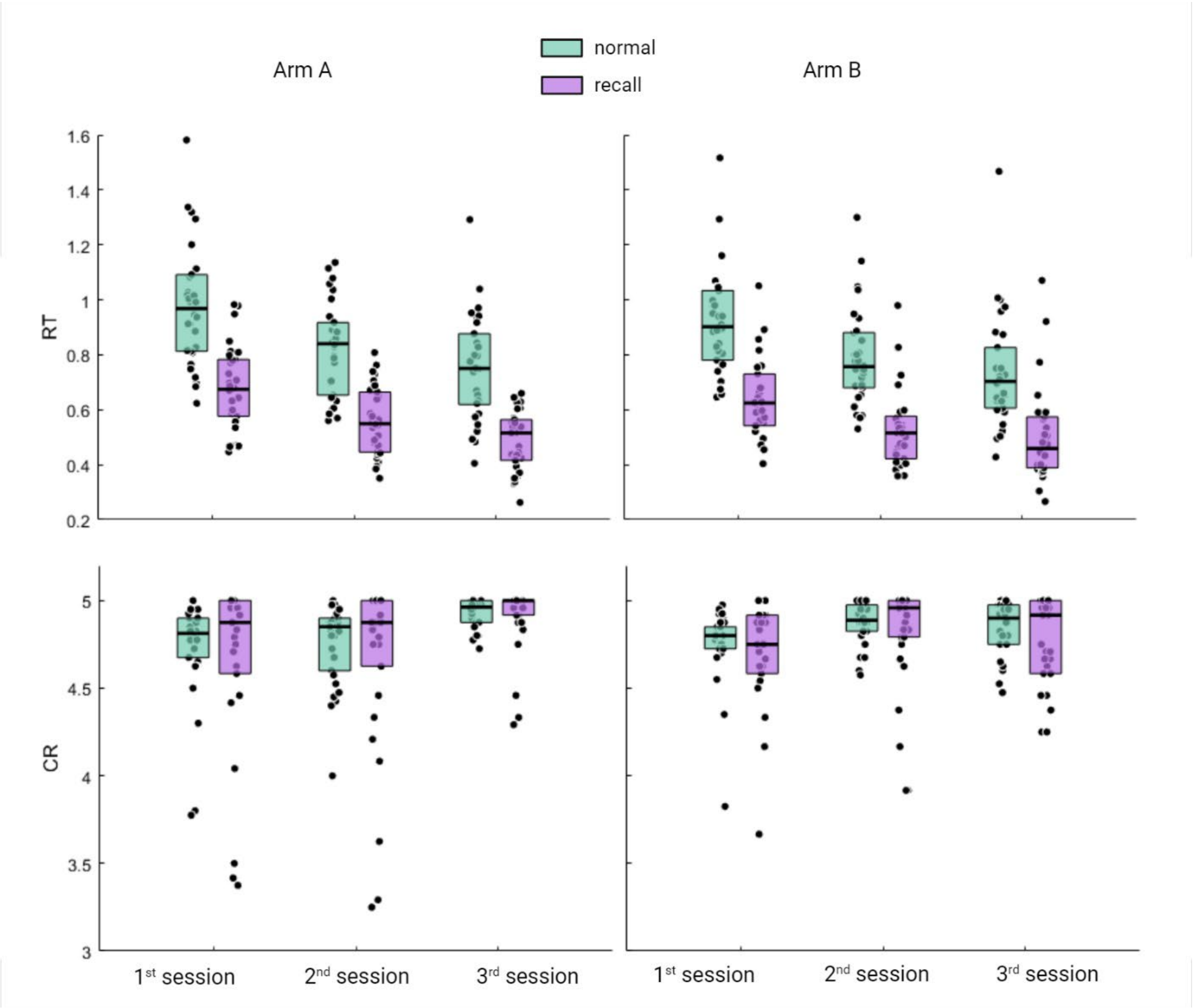

Figure S2. Comparing task scores under normal and recall task conditions among young healthy volunteers. Boxplots depicting the median, the 25th and 75th quartile values of of Reaction Time (RT ;the time in seconds it takes to complete key press, upper panel) and Correct Responses (CR; number of correct responses per five-element sequence) averaged under normal conditions (green bars) and under recall conditions (purple bars) across the three task sessions of arm A (left panel) and arm B (right panel). Individual data points are plotted as black circles.

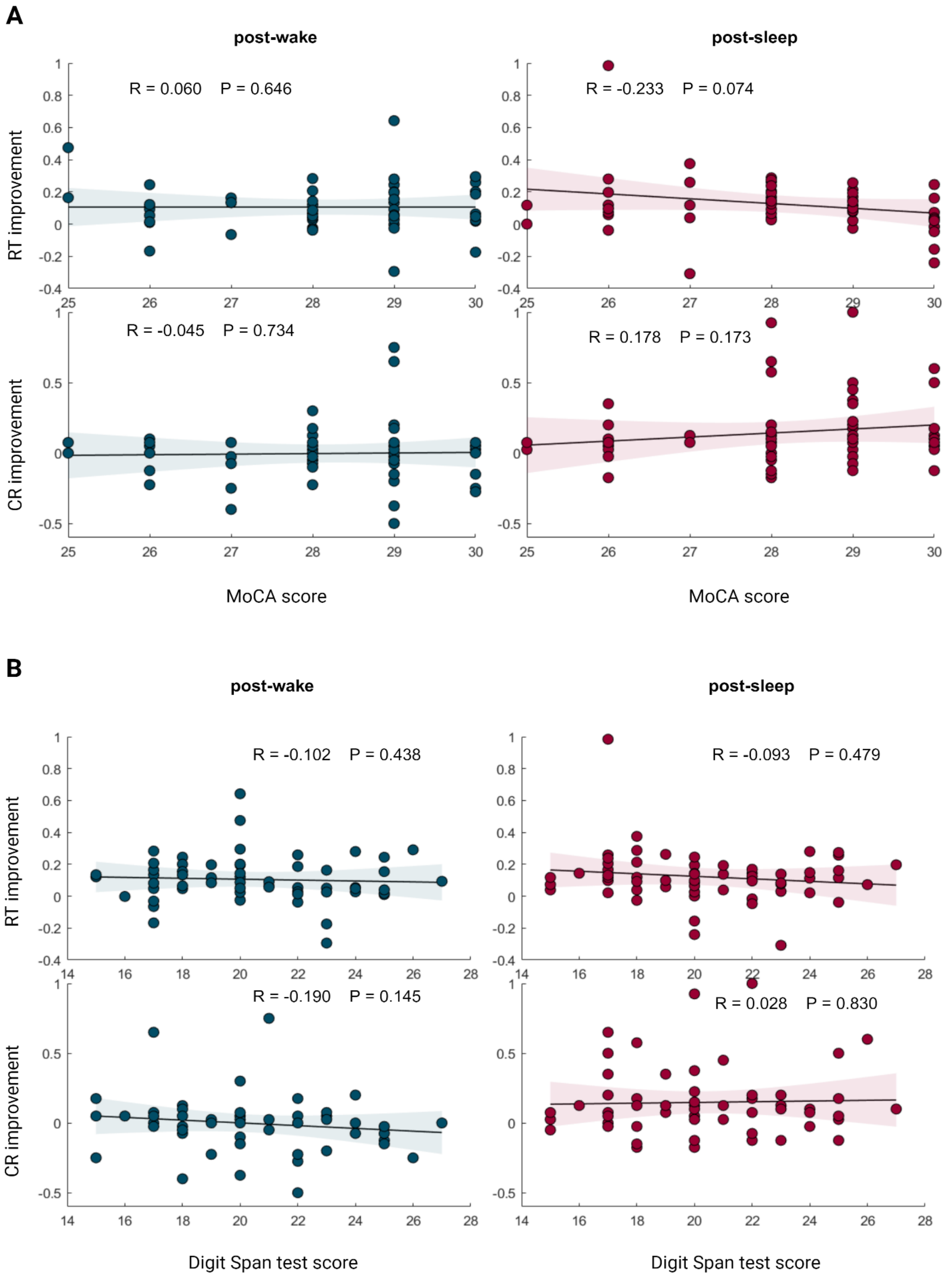

Figure S3. Correlation between task improvement and cognitive assessment scores of young

participants following wake and sleep intervals. Individual improvement indices were calculated as the difference between the mean scores of two consecutive sessions, separated by either sleep or wake intervals (i.e., post-sleep [maroon] or post-wake [turquoise], respectively). These indices were combined across participants from both arms for RT (upper panel) and CR (lower panel). Individual indices are represented as circles, with the solid black line showing the linear regression plotted against the cognitive assessment scores, and the shaded area representing the 97.5% confidence interval. **(A)** Spearman's rank correlation test for task improvement indices versus MoCA (Montreal Cognitive Assessment) scores. **(B)** Spearman's rank correlation test for task improvement indices versus Digit Span test total scores. The p-value cutoff, following adjustment for multiple comparisons, is 0.025. Task improvement was not significantly correlated with either the MoCA score or the Digit Span score.

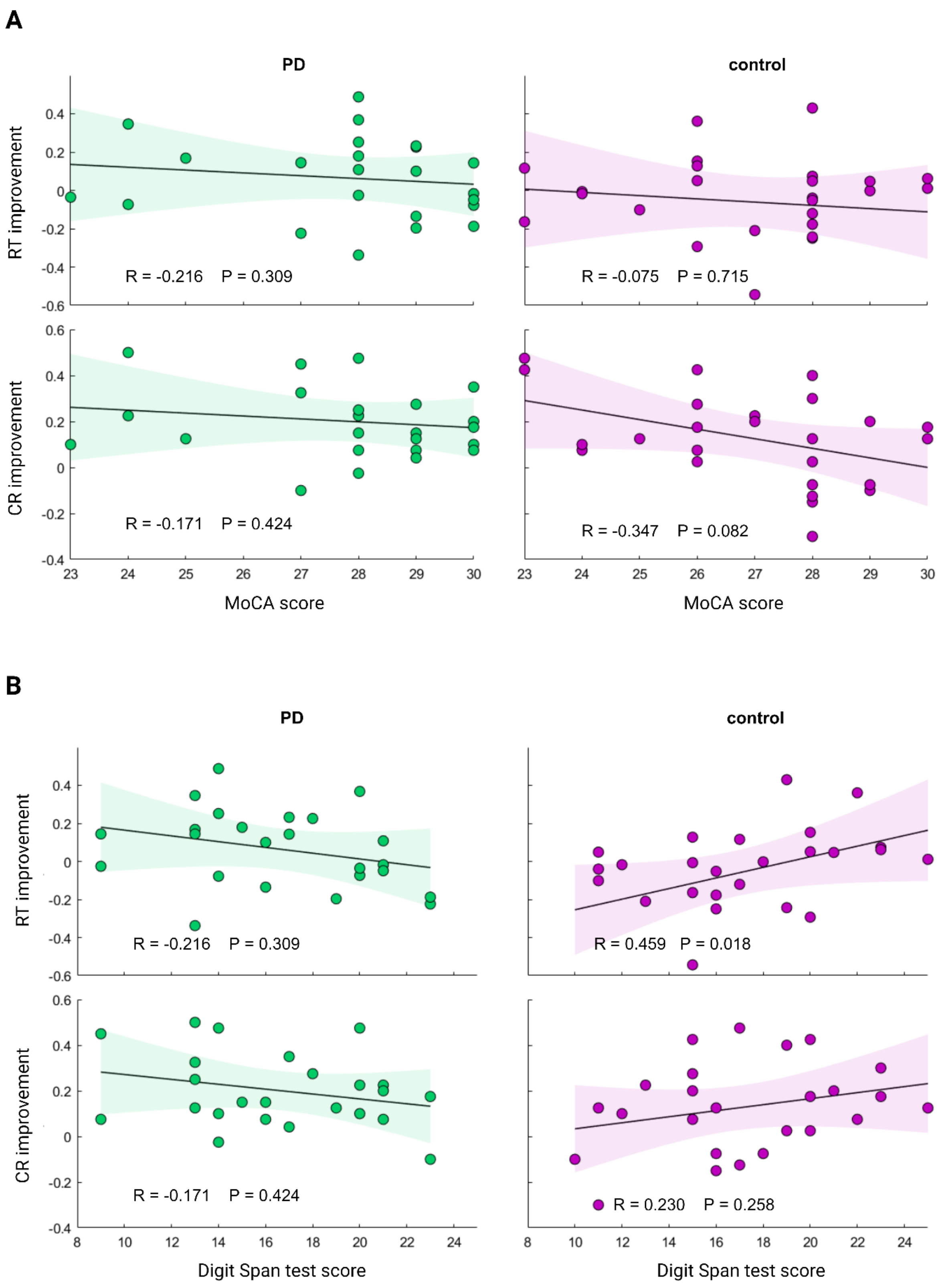

Figure S4. Correlation between post-sleep task improvement and cognitive assessment scores among

patients with Parkinson's Disease (PD) and healthy individuals of comparable age. Individual improvement indices were calculated as the difference between the mean scores of two consecutive sessions, separated by a sleep interval. These indices are plotted for participants from the control group (green, left panel) and the PD group (purple, right panel) for both RT (upper panel) and CR (lower panel). Individual indices are represented as circles, with the solid black line showing the linear regression plotted against the cognitive assessment scores, and the shaded area representing the 97.5% confidence interval. **(A)** Spearman's rank correlation test for task improvement indices versus MoCA (Montreal Cognitive Assessment) scores. **(B)** Spearman's rank correlation test for task improvement indices versus Digit Span test total scores. The p-value cutoff, following adjustment for multiple comparisons, is 0.025 in the control group and 0.0125 in the PD group (due to additional comparisons against motor symptom severity). In both groups, task improvement was not significantly correlated with either the MoCA score or the Digit Span score.

1.2 Supplementary Tables

Table S1. Differences in performance under normal and recall task conditions.

		Arm A			Arm B		
		1st session	2nd session	3rd session	1st session	2nd session	3rd session
RT	Difference (%)	29.8 ± 12.3	31.8 ± 9.6	33.7 ± 8.7	31.1 ±14.8	34.9 ± 14.0	33.5 ± 16.4
	Z- statistic	4.78	4.78	4.78	4.78	4.78	4.78
	P-value	0.000	0.000	0.000	0.000	0.000	0.000
CR	Difference (%)	1.5 ± 6.5	1.8 ± 9.1	0.6 ± 4.3	0.9 ±7.1	1.5 ± 5.8	1.4 ± 6.3
	Z- statistic	0.62	0.11	-0.71	-0.13	0.28	1.15
	P-value	0.538	0.914	0.474	0.894	0.782	0.252

The percentage of changes between normal and recall conditions was calculated for individual subjects [$\frac{normal-recall}{normal} \times 100\%$] and averaged for each arm across task sessions. Results are presented as mean ± standard deviation. The Wilcoxon signed-rank test was used for further analysis.
