# Supplementary figures and images for: Sleep improves accuracy, but not speed, of generalized motor learning in young and older adults and in individuals with Parkinson’s disease
Source: Front Behav Neurosci. 2024 Sep 26;18:1466696. doi: 10.3389/fnbeh.2024.1466696 (PMC11464313; doi:10.3389/fnbeh.2024.1466696)

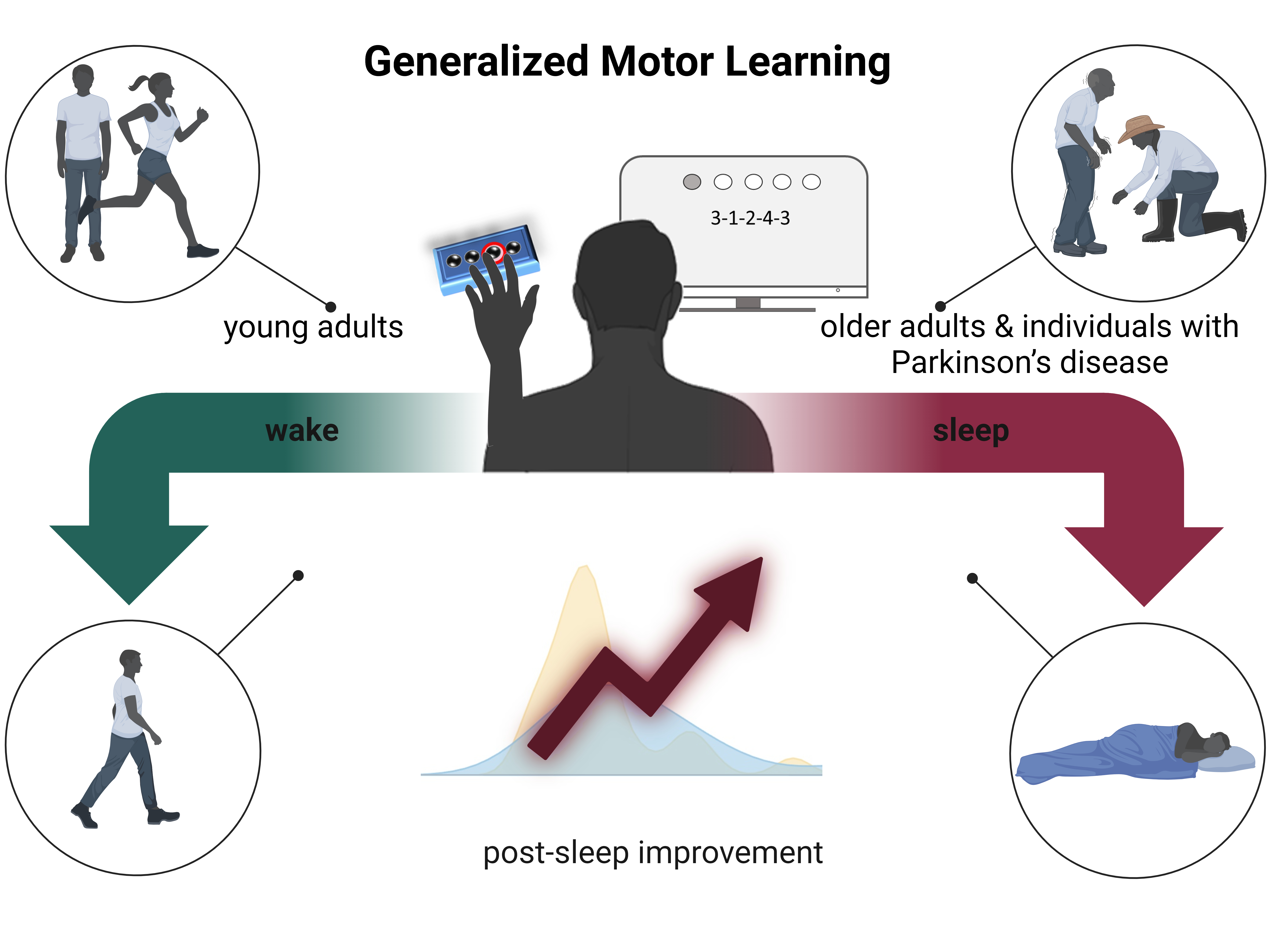

Supplement: Supplementary file 3 [file Image_1.JPEG]
